# Performance Enhancement of Interdigital Electrode-Piezoelectric Quartz Crystal (IDE-PQC) Salt Concentration Sensor by Increasing the Electrode Area of Piezoelectric Quartz Crystal (PQC)

**DOI:** 10.3390/s18103224

**Published:** 2018-09-25

**Authors:** Hui Zhang, Yao Yao, Yue Shi

**Affiliations:** 1 College of Communication Engineering, Chengdu University of Information Technology, Chengdu 610225, China; symxmyzhang@163.com (H.Z.); october@cuit.edu.cn (Y.S.); 2 School of Automation Engineering, University of Electronic Science and Technology of China, Chengdu 610054, China; 3 Engineering Research Center for Electronic Microsystems, Chengdu University of Information Technology, Chengdu 610225, China

**Keywords:** salt solution concentration sensor, IDE-PQC, sensitivity enhancement, frequency stability

## Abstract

In this paper, a new approach to enhance the performance of the interdigital electrode-piezoelectric quartz crystal (IDE-PQC) salt solution concentration sensor by modifying the electrode area of PQC was proposed. Equivalent circuit analysis showed that the static capacitor (*C*_0_) which is related to the electrode area of PQC directly affected the response sensitivity of the IDE-PQC sensor. Further, the sensing responses of IDE-PQC sensors to various concentrations of salt solution were measured. Three kinds of salt solution, including NaCl, KCl, and Na_2_SO_4_, were adpoted to evaluate the sensing performances of the IDE-PQC sensors. The experimental results also indicated that increasing the electrode area of PQC can enhance the sensitivity response of the IDE-PQC sensors to the change of salt solution concentration. For example, the detection sensitivity of the IDE-PQC sensor with an electrode diameter of 5 mm was about three times larger than that of the sensor with an electrode diameter of 3 mm. Meanwhile, we found that the frequency stability of the IDE-PQC sensor was also improved by increasing the electrode area of PQC. In addition, the influence of the electrode area of PQC on the repeatability and the transient response of IDE-PQC salt solution concentration sensor were also studied. This work demonstrates simple and cost-effective method to achieve the performance enhancement of IDE-PQC salt solution concentration sensor by modifying the electrode area of PQC.

## 1. Introduction

The accurate measurement of salt solution concentration plays a vital role in many engineering fields, such as food, medicine, industrial automation and environmental protection [1,2]. Up to now, a large variety of methods for detecting salt solution concentration in liquid, such as ion-sensitive electrode [3], optical fiber [4,5], capacitively coupled contactless conductivity detection (C^4^D) [6], electromagnetic [7,8], quartz crystal microbalance (QCM) [9], and series piezoelectric quartz crystal (SPQC) [10] techniques, have been reported in the literatures. Among them, both QCM and SPQC techniques have used piezoelectric quartz crystal (PQC) as a basic element. In many applications, QCM is used as a mass sensitive platform, according to the Sauerbrey equation [11]. Some recent studies have provided numerical models to analyze the mass-sensitivity distribution of the QCM as a function of its electrode area [12] and demonstrated the importance of sensor surface’s roughness for the adjustment of the QCM’s sensitivity in gas and liquid environments [13,14,15]. However, the sensitive mechanisms of QCM and SPQC for detecting salt solution concentration are not based on the mass-sensitivity of QCM. As reported by Ref [9], one electrode of QCM contact with analyte solution. In this case, QCM is used as sensitive element, and the sensitive mechanism is based on the electrical interaction between the fringing electric field that is formed by the electrodes of QCM and analyte solution, as well as the mechanical interaction between the QCM electrode and analyte solution. The electrical interaction mentioned above makes QCM produce a frequency shift related to the change of the conductivity and dielectric constant of analyte solution, while the mechanical interaction makes QCM yield a frequency shifts related to the change of the density and viscosity of analyte solution. It is noteworthy that the contact between QCM electrode and analyte solution will weaken the quality factor of QCM due to the increase of damping, resulting in the decrease of frequency stability [16]. SPQC technique which was first reported by S. Yao has attracted considerable attentions due to its advantages of simple circuit structure, frequency signal output, high stability, on-line measurement, and low cost [10]. The SPQC sensor is generally constructed by connecting a PQC and a conductivity electrode in series. In practical applications, the conductivity electrode, which is used as sensitive element is immersed into the analyte solution during the measurement. The SPQC sensor can change the slightly change in resistance or capacitance of conductivity electrode to a frequency shift. Because PQC used here does not need to contact with analyte solution, it can be sealed by a metal shell and filled with inert gas. This enables PQC to have high quality factor (QF) as high as 10^5^~10^6^. Thus the SPQC sensor can provide high frequency stability. As a pioneer researcher in this field, S. Yao firstly reported the application of SPQC sensor for end-point determination in frequencimetric titrations [17]. Since then, his group reported a large amount of application of SPQC in analytical chemistry, such as the activity of urease in plant seeds and BOD of microorganism metabolism [18,19].

Although the SPQC sensor provides sensitive ability to the change in solution concentration in liquid, there has been still a great challenge to enhance the detection sensitivity of the sensor. To address this issue, researchers attempted to improve the detection sensitivity of the SPQC sensor through modifying its structure. For example, He et al. reported a new interdigital electrode (IDE)-PQC sensor that adopted IDE electrodes instead of conventional conductivity electrodes to connect with the PQC, and experimentally observed that IDE-PQC sensor is more sensitive than SPQC to the change in solution concentration in liquid [20]. Besides the modification of conductivity electrode, the characteristic parameter of PQC has also been found to determine the sensitivity of IDE-PQC sensor. For instance, the sensitivity of IDE-PQC sensor can be improved by increasing the fundamental resonance frequency of PQC [21]. This method is simple and convenient since it just needs to replace the PQC device with different fundamental frequency. However, J. Vig also emphasized that a higher-frequency PQC can lead to lower accuracy and to a lesser ability to resolve small variations in the measurement [21]. In addition, a higher-frequency PQC results in the disadvantages of a higher aging rate and larger power consumption. Although there are limitations to enhance sensitivity by increasing the fundamental frequency of PQC, it provides a beneficial guideline to realize the tunable sensitivity of IDE-PQC sensor by adjusting the parameter of PQC.

Besides the fundamental resonance frequency, the electrode area of PQC is also an important characteristic parameter to determine the mass sensitivity of PQC according to the Sauerbrey equation [11]. However, to our best knowledge, few attentions were paid on the affect of the electrode area on the performance of the IDE-PQC sensor. Thus, the specific objective of this study is to explore the influence of the electrode area of PQC on the sensing performance of IDE-PQC salt solution concentration sensor. Firstly, the mechanism of the tunable sensitivity of IDE-PQC sensor by adjusting the electrode area of PQC is analyzed by equivalent circuit method. Secondly, various performance characteristics of IDE-PQC sensors with three different electrode areas, including detection sensitivity, frequency stability, repeatability, and transient response, are experimentally investigated and compared.

## 2. Experimental

**Figure 1:**
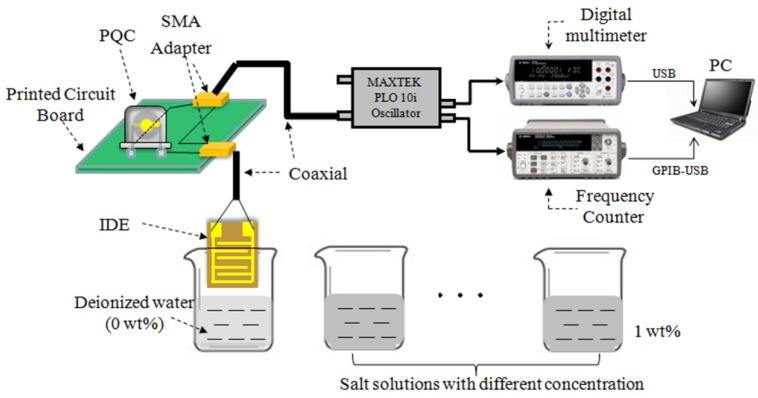
Schematic diagram of the experimental setup.

**Figure 2:**
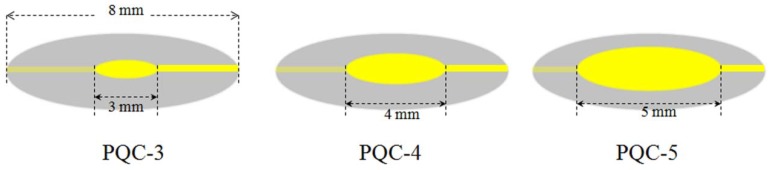
Illustration of the fabricated piezoelectric quartz crystal (PQCs) with three kinds of electrode diameters.

Figure 1 shows the schematic diagram of the whole experimental setup. It is mainly composed of the following four parts: sensing unit, exciting circuit unit, data acquisition and processing unit and various salt solutions with different concentration. The sensing unit is IDE-PQC structure, it consists of an IDE and a PQC connected in series. The IDEs were predesigned and fabricated directly on a thin flexible Polyethylene terephthalate (PET) substrate using the Flexible Printed Circuit Board (FPCB) process. Polyethylene terephthalate (PET) is chosen as the substrate of IDE due to its advantage of flexibility and low cost. Au/Ni/Cu multilayer electrode was successively deposited on the PET substrate using the electrochemical deposition method. The geometric parameters of IDE are 1 cm × 1 cm in size and 200 μm in electrodes separation. The electrodes were patterned by a photo/etching process. The PQCs with two identical silver circular electrodes being deposited on both sides of quartz disk are designed and fabricated by Wuhan Hitrusty Electronics. Multiple PQCs with different diameters of silver electrode are fabricated to study the influence of the electrode area on the performance of IDE-PQC sensor. In this work, the diameter of quartz disk is 8 mm, and three kinds of diameters of silver electrode (5, 4 and 3 mm) are employed. The PQCs that are used in this work are sealed by a metal shell and filled with inert gas in order to provide high QF. The fabricated PQCs are labeled as PQC-5, PQC-4 and PQC-3 as shown in Figure 2, respectively. The electrically exciting circuit unit adopts a commercial phase-locked loop oscillator (Plo10i, Maxtek Inc., Santa Fe Springs, CA, USA), which can excite the IDE-PQC to produce oscillation and provide frequency signal and resonance resistance (*R*_r_) information. The series resistance (*R*_s_) is usually used to evaluate the frequency stability of IDE-PQC sensor. The smaller the series resistance is, the higher the frequency stability. The data acquisition and processing unit includes a frequency counter (53131A, Agilent Technologies, Santa Clara, CA, USA), a digital multimeter (34410A, Agilent Technologies, Santa Clara, CA, USA) and a PC. The frequency counter is used to monitor the frequency of IDE-PQC sensor, and the digital multimeter is adopted to measure the series resistance of IDE-PQC sensor. Three kinds of salt, including NaCl, KCl and Na_2_SO_4_, are selected for evaluating the sensing performance of the sensor. The salt solutions with different concentrations are obtained by dissolving salt in deionized water, and the deionized water with a resistivity of 18.24 MΩ·cm is treated as zero concentration. The chemicals are analytical grade and are used without further purification. All of the experiments are carried out at 25 °C without special instructions.

## 3. Results and Discussion

**Figure 3:**
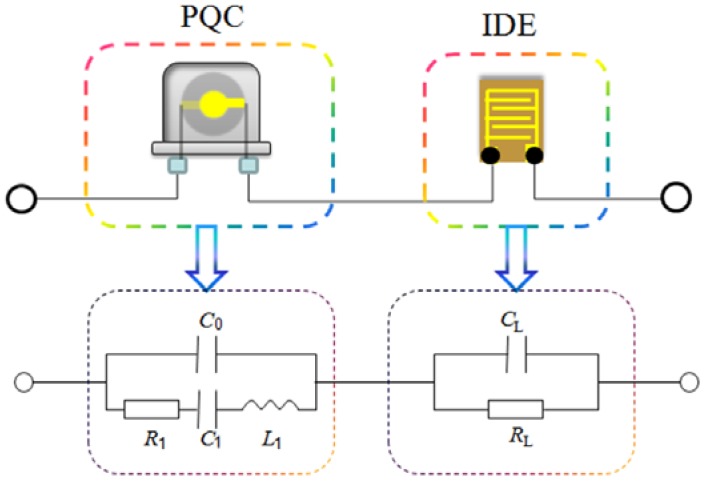
Equivalent circuit model of Interdigital Electrode-Piezoelectric Quartz Crystal (IDE-PQC) sensor.

**Figure 4:**
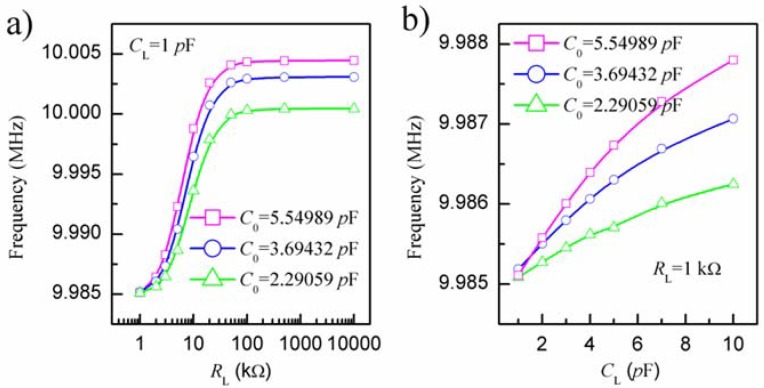
Simulation result of the resonance frequency of IDE-PQC sensor as a function of (**a**) *R*_L_ or (**b**) *C*_L_.

Electrical equivalent circuit model, as shown in Figure 3, is a very useful tool to describe and analyze the physical behaviors of PQC [22] and IDE [23]. The electrical equivalent circuit of PQC is composed of a series motional branch, including a capacitor (*C*_1_), an inductor (*L*_1_), and a resistor (*R*_1_), and a parallel static branch, including a capacitor (*C*_0_) [22]. The series motional branch (*R*_1_, *L*_1_ and *C*_1_) is associated with the vibrating of quartz plate, *R*_1_ represents the energy dissipation during oscillation due to internal friction, mechanical losses in the mounting system and acoustical losses to the surrounding environment, *L*_1_ describes the initial quality of quartz crystal, *C*_1_ represents the energy stored during oscillation [24]. The parallel static branch (*C*_0_) is related to the electrode area across the quartz disk. The electrical equivalent circuit of IDE can be viewed as a resistor (*R*_L_) and a capacitor (*C*_L_) connected in parallel [23]. When IDE is immersed in the salt solution for concentration measurement, the element *R*_L_ and *C*_L_ are associated with the conductivity and dielectric constant of solution. By using the Agilent ADS simulator, we simulated the resonance frequency of IDE-PQC sensor as a function of *R*_L_ or *C*_L_ using the above electrical equivalent circuit model. Table 1 lists the typical electrical equivalent circuit parameters of 10 MHz PQCs with different diameters of silver electrode being used in this experiment. It can be especially found that the *C*_0_ depends on the diameter of silver electrode. The simulation results are shown in Figure 4. From the graph we can easily see that the frequency response of IDE-PQC sensor to the change of *R*_L_ or *C*_L_ is related to the *C*_0_. The IDE-PQC sensor with larger *C*_0_ exhibited larger frequency shifts. Besides the electrical equivalent circuit simulation, the frequency response sensitivities, $\frac{\partial f}{\partial C_{L}}$, have also been derived by using the electrical equivalent circuit model, as shown in Figure 3. Using the equivalent method similar as X. Huang [25], we can obtain the sensitivity expression, as follows:

(1) $$\frac{\partial f}{\partial C_{L}}\approx -f_{0}\frac{\chi C_{1}}{2{\left(C_{0}+C_{L}\right)}^{2}}$$

where *f* is the series resonance frequency of equivalent circuit network shown in Figure 3, *f*_0_ is the series resonance frequency of PQC, and *χ* is constant. From this equation, we can also find that the *C*_0_ value influence the frequency response of IDE-PQC sensor to the change of *C*_L_.

**Figure 5:**
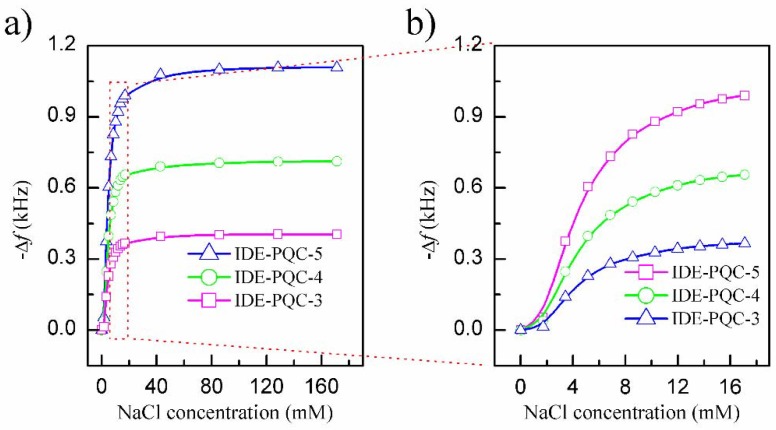
Frequency shifts of IDE-PQC sensor at different NaCl solution concentration.

Next, the sensing experiments were carried out by immersing IDE into various NaCl solution concentrations successively, and the resonance frequency of IDE-PQC sensor was recorded. The NaCl solution concentration was varied from 0 to 171.1 mM. Figure 5 presents the measured resonance frequency shifts of the IDE-PQC sensors at different NaCl solution concentration. From this figure, we can see that all the IDE-PQC sensors with different electrode diameters display the same response tendency, and the resonance frequency of the IDE-PQC sensor decreases monotonically as increasing the NaCl solution concentration from 0 to 17.1 mM. Such frequency shifts can be used to determine the unknown NaCl solution concentration. Moreover, we note that the frequency response of IDE-PQC sensors reaches saturation at the concentration of 17.1 mM and upon further increase in the NaCl solution concentration, the generated frequency shifts become relatively constant. This result is in accord with Yao’s work indicating that the IDE-PQC sensor is more suitable for the detection of low concentration salt solution system [10]. More importantly, it is noteworthy that the detection sensitivity of IDE-PQC sensor is apparently affected by the electrode area of PQC. In particular, the maximum frequency shifts of the IDE-PQC-5 is 991 Hz as the NaCl solution concentration changes from 0 to 171.1 mM, which is about three times larger than that of the IDE-PQC-3. This experimental result is consistent with the simulation results that are described above. The electrode area of PQC directly determines its static capacitance *C*_0_ according to the well-known parallel plate capacitance theory. Hence, the IDE-PQC sensor with a larger electrode has a larger *C*_0_ value, finally resulting in higher response sensitivity to the change of NaCl solution concentration.

**Figure 6:**
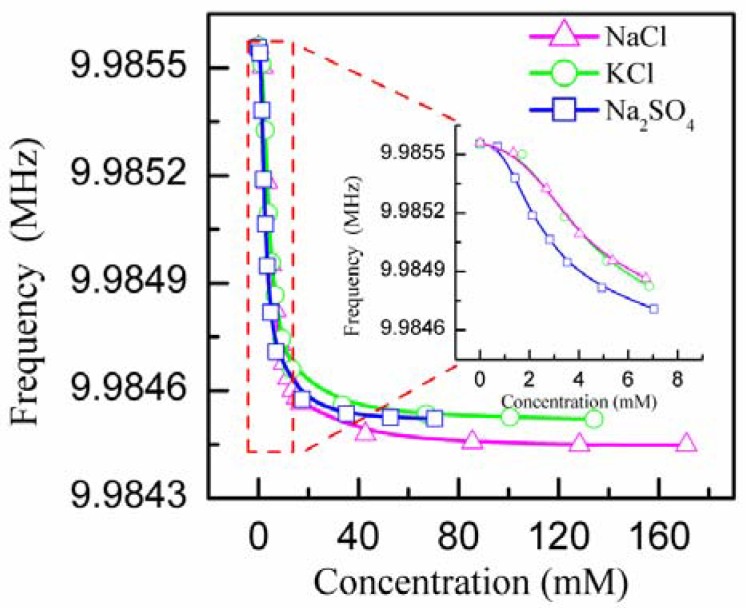
Frequency shifts of the IDE-PQC-5 sensor to different salt solutions.

Besides NaCl solution, we have also measured the frequency response of IDE-PQC sensor to other salt solutions, such as the KCl and Na_2_SO_4_ solution. The frequency response curves of the IDE-PQC-5 sensor are plotted in Figure 6. The inset of Figure 6 shows the frequency shifts of IDE-PQC-5 sensor in a low concentration range. As is seen, the response trend of IDE-PQC-5 sensor to different salt solutions is basically similar. The frequency of the IDE-PQC-5 sensor decreases monotonically while increasing the solution concentration. Moreover, it can be also found that the frequency response of IDE-PQC-5 sensor to Na_2_SO_4_ solution is larger than that of NaCl or KCl solution, and the frequency response of the sensor to NaCl solution is almost equal to that of KCl solution. The observed difference in frequency response of sensors to three kinds of salt solutions can be mainly attributed to the amount of ions. Since Na_2_SO_4_ provides the largest amount of ions at the same molar concentration among the three kinds of salts, the frequency shift of the sensor to Na_2_SO_4_ in the low concentration range is the largest. The same reason can be used to explain the equal sensor’s signals yielded by NaCl and KCl solution. This result demonstrates that IDE-PQC sensor can be used to measure the concentration of the known salt solution in a low concentration range.

**Figure 7:**
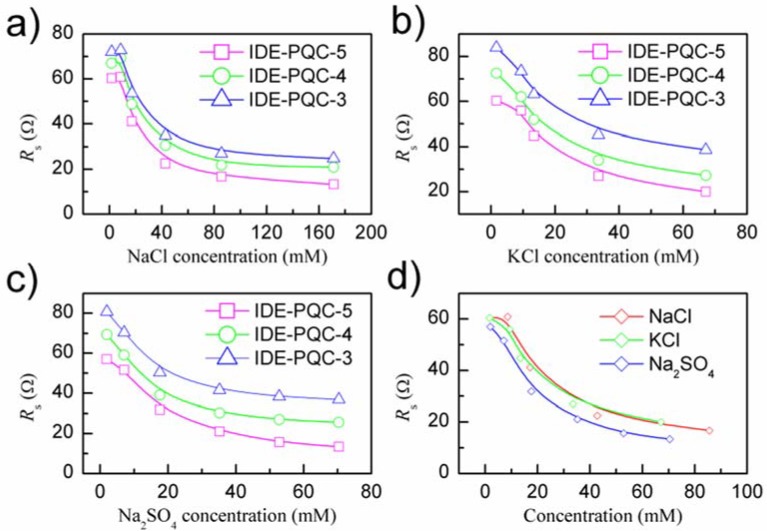
The series resistance *R*_s_ of IDE-PQC sensor at different concentrations of (**a**) NaCl. (**b**) KCl and (**c**) Na_2_SO_4_ solution, and (**d**) The comparison of the series resistance *R*_s_ of IDE-PQC-5 sensor at different salts solution.

As is well known, the frequency stability is also a very important parameter to evaluate the performance of IDE-PQC sensor, especially when the sensor operates in a liquid environment. The series resistance (*R*_s_), which is the resistance component in the motional branch of BVD equivalent electrical circuit is used to describe the frequency stability of IDE-PQC sensor. This parameter determines the energy losses per wave cycle and hence, the quality factor of the resonator. High *R*_s_ value indicates low quality factor and low frequency stability. Figure 7a–c gives the curves of the series resistances of IDE-PQC sensors during salt concentration measurement, and Figure 7d plots the comparing curve of the series resistance of IDE-PQC-5 sensor for different salt solutions. As can be seen in Figure 7a–c, the series resistance of IDE-PQC sensors decreases with increasing the salt solution concentration for all the three kinds of salt solutions. The reason for the above-described phenomena can be explained by the fact that the increase of salt solution concentration increases the conductivity of the aqueous solution, i.e., decreases the series resistance. Meanwhile, it is clearly seen through the experiment that the series resistance of IDE-PQC sensors in the entire detection range is below 90 Ω. Such low series resistance can ensure that the IDE-PQC sensor has high frequency stability during the concentration measurement. Furthermore, the curves that are shown in Figure 7a–c also indicate the characteristic that the electrode area of PQC affects the frequency stability of IDE-PQC sensor. It can be found that the IDE-PQC-5 sensor has the smallest series resistance among the three sensors. This is due to the fact that the electrode area *A* of PQC determines the resistance element *R*_1_ shown in Figure 3 according to Equation (2) [24]. So, the increase of the electrode area *A* of PQC decreases the resistance element *R*_1_, further decreases the series resistance *R*_s_ of IDE-PQC sensor. This finding indicates that the IDE-PQC sensor with larger electrode area also provides higher frequency stability during the concentration measurement. Summarizing the above-described experimental results, we can conclude that increasing the electrode area of PQC can improve both the detection sensitivity and the frequency stability of the IDE-PQC sensor.

(2) $$R_{1}=\frac{t_{q}^{3}r}{8A\varepsilon^{2}}$$

**Figure 8:**
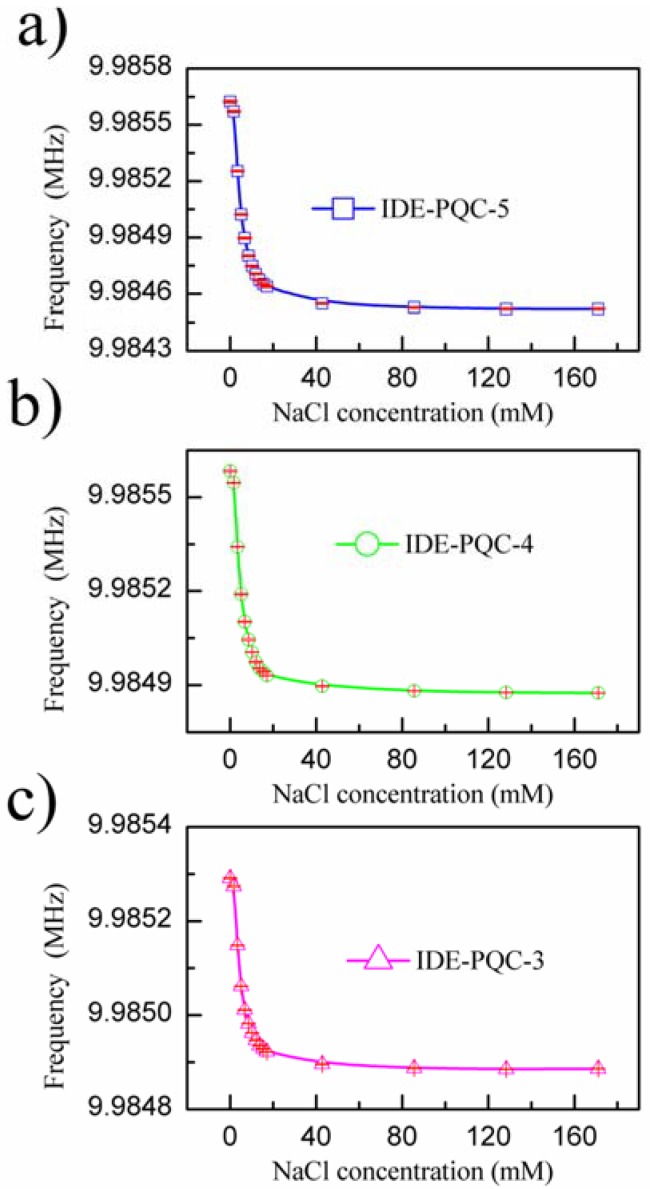
The response with error bar of IDE-PQC sensor. The error bar represents standard deviation from the mean.

**Figure 9:**
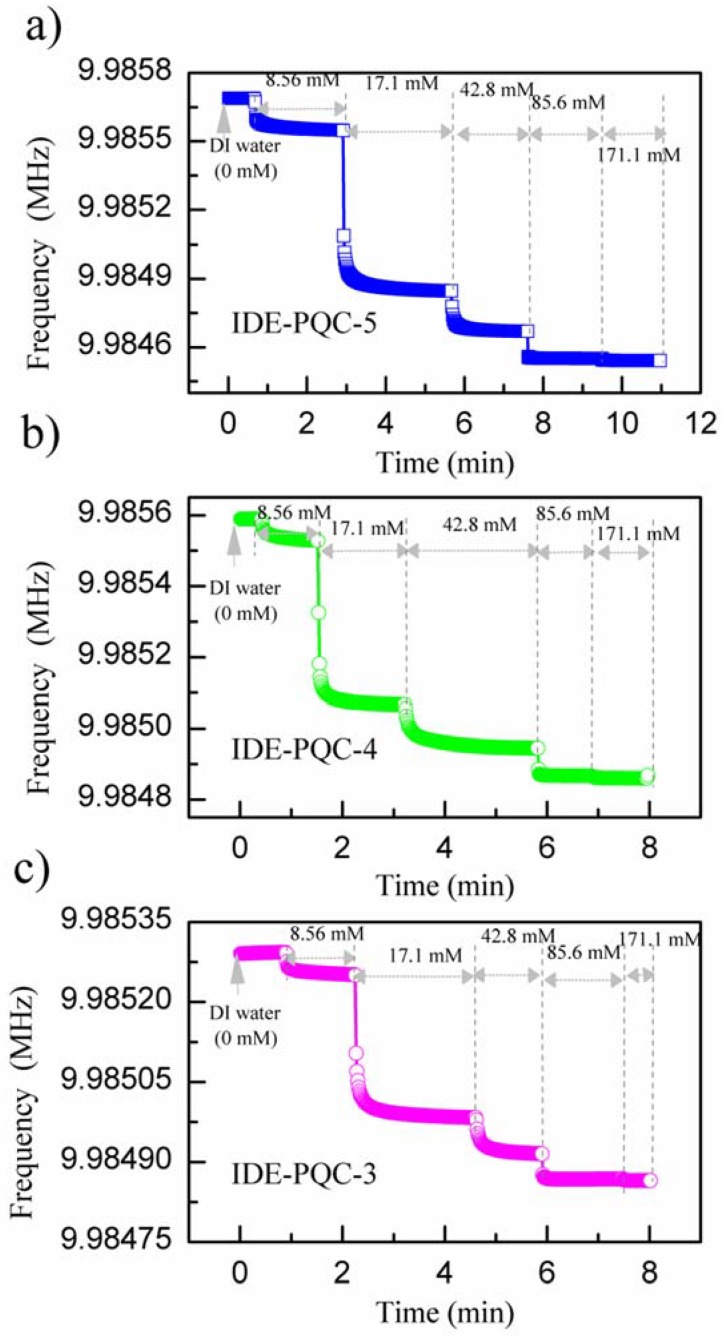
Transient response of IDE-PQC sensor to different NaCl solution concentrations.

The repeatability is also vital for the salt concentration sensor. To study the repeatability of the IDE-PQC sensor, we measured the frequency shifts of IDE-PQC sensors to the change of concentration ranging from 0 to 171.1 mM for three times under identical experimental conditions. The record data are plotted to show the response difference between three test procedures. Figure 8 shows the repetitive responses with an error bar of IDE-PQC sensor. The error bar represents standard deviation (SD) from the mean. It can be found that the error of sensors is small during multiple tests, which indicates that the IDE-PQC sensor has excellent repeatability. The excellent repeatability that is provided by the IDE-PQC sensor may be attributed to the following two aspects: one is the unique detection method of the IDE-PQC sensor; the other is high stability of PQC. Regarding to the detection method of the IDE-PQC sensor, only IDE is immersed in the liquid and acted as sensing element when the IDE-PQC sensor is used for liquid concentration measurement, in this case, the change in the conductivity or dielectric constant of the liquid can only cause the IDE to produce resistance or capacitance changes. Importantly, the change in some other properties of the solution, such as density and viscosity, do not affect the PQC. Additionally, it is noticed that PQC does not need to immerse in the solution during measurement; as a result, the inherent high quality factor of PQC can provide high frequency stability for the IDE-PQC sensor. In addition to the above experiment results, the transient response of IDE-PQC sensor was also measured by successively immersing the IDE to several fixed concentrations of NaCl aqueous solution. The transient response curves are illustrated in Figure 9. From the graph, we can see that the IDE-PQC sensor exhibits a desired step response toward various concentrations of NaCl aqueous solution. This result indicates that the IDE-PQC sensor possess fast response to the change of salt concentration. Meanwhile, it can be also found that the increase of the electrode area of PQC does not sacrifice the transient response performance of the IDE-PQC sensor.

## 4. Conclusions

In summary, the present study systematically studied the influence of the electrode area of PQC on the sensing performance of IDE-PQC salt concentration sensor. The electrical equivalent circuit model was established to analyze the sensitive enhancement mechanism of IDE-PQC salt concentration sensor. Simulation and experimental results proved that the sensing performance of IDE-PQC salt concentration sensor was associated with the electrode area of PQC. Increasing the electrode area of PQC, on the one hand, can increase the detection sensitivity of IDE-PQC salt concentration sensor; and, on the other hand, it can also improve the frequency stability of IDE-PQC salt concentration sensor. Moreover, the IDE-PQC salt concentration sensor exhibited an excellent repeatability and a fast response to the change of salt concentration. This work provides a simple and cost-effective method to achieve the sensitivity and stability enhancement of IDE-PQC salt concentration sensor by modifying the electrode area of PQC.

## Figures and Tables

**Table 1:** The equivalent circuit parameters of 10 MHz PQCs with different diameters of silver electrode.

The Label of QCM	*R*_1_ (Ω)	*C*_1_ (*f*F)	*L*_1_ (*m*H)	*C*_0_ (*p*F)	QF
PQC-5	9.3141	25.9809	9.77955	5.54989	48293
PQC-4	30.8075	17.0796	14.8755	3.69432	47518
PQC-3	10.0523	10.2552	24.7747	2.29059	39387
